# Association of the *LILRA3* Deletion with B-NHL and Functional Characterization of the Immunostimulatory Molecule

**DOI:** 10.1371/journal.pone.0081360

**Published:** 2013-12-09

**Authors:** Hui Zhi Low, Sandra Reuter, Michael Topperwien, Nadine Dankenbrink, Dietrich Peest, Gamze Kabalak, Renata Stripecke, Reinhold E. Schmidt, Torsten Matthias, Torsten Witte

**Affiliations:** 1 Clinic for Immunology and Rheumatology, Hannover Medical School, Hannover, Germany; 2 Department of Hematology, Hemostasis, Oncology and Stem Cell Transplantation, Hannover Medical School, Hannover, Germany; 3 AESKU.KIPP Institute, Wendelsheim, Germany; 4 AESKU Diagnostics, Wendelsheim, Germany; Pontificia Universidade Catolica do Rio Grande do Sul, Brazil

## Abstract

LILRA3 is the sole soluble member of the LILR family. Previous studies from our group had shown that a 6.7 kb genetic deletion of *LILRA3* is associated with MS and Sjögren’s syndrome. An impairment of the immune response leads to a predisposition for B-NHL, so we wanted to study whether the deletion of *LILRA3* is also a risk factor for B-NHL, as well as the function of LILRA3. We discovered that the frequency of the homozygous *LILRA3* deletion was significantly higher in B-NHL (6%) than in blood donors (3%) (P = 0.03). We detected binding of fluorochrome-conjugated recombinant LILRA3 to monocytes and B-cells. Incubation of PBMCs with recombinant LILRA3 induced proliferation of CD8^+^ T-cells and NK cells, as determined by CFSE staining. Using a transwell system, we demonstrated that LILRA3-stimulated lymphocyte proliferation was mediated by monocytes and required both cell contact and soluble factors. Secretion of IL-6, IL-8, IL-1β and IL-10 in the cell supernatant was stimulated by LILRA3. We conclude that LILRA3 is an immunostimulatory molecule, whose deficiency is associated with higher frequency of B-NHL.

## Introduction

The leukocyte-immunoglobulin (Ig)-like-receptors (LILRs) are a family of receptors located within the leukocyte receptor cluster on chromosome 19q13.4, in the vicinity of the killer cell inhibitory receptors and the leukocyte associated inhibitory receptors [1]–[3]. Members of the LILR family have extracellular regions consisting of 2 or 4 Ig-like C2-type domains [4] and their cytoplasmic region determines whether they play activating or inhibiting roles. LILRs shown to be inhibitory, such as LILRB1 (CD85j; ILT2; LIR-1), have long intracellular regions containing immunoreceptor tyrosine-based inhibitory motifs (ITIMs) [5], [6]. On the other hand, activating LILRs like LILRA4 (ILT7) and LILRA2 (CD85h; ILT1), have short intracellular regions and an arginine residue located within the transmembrane region which allows for coupling to activating adaptor molecules like FcεRIγ [7], [8]. LILRA3 (CD85e; ILT6) corresponds to a less characterized member of the family, lacking transmembrane or cytoplasmic regions, and thus existing solely as a soluble protein [5], [9], [10].

Unlike the killer cell inhibitory receptors, genetic polymorphisms in the LILR family are quite rare, with the exception of *LILRA3* which can exist as a deletion genotype [11], [12] and *LILRB3* whereby 15 different variants have been described [13] and that the different variants were able to induce an antibody response to LILRB3 in *LILRB3*-mismatched hematopoietic stem cell transplations [14]. Among the Caucasians, the homozygous deletion genotype is present in 3–5.5% of the healthy population, and has been shown to be associated with multiple sclerosis and Sjögren’s syndrome [6], [15], [16]. On the other end of the immune disorder spectrum, whether *LILRA3* deletion could affect the risk of developing malignant diseases is as yet unknown. B-non-Hodgkin’s lymphoma (B-NHL) is a diverse group of B-cell malignancies, for which the most pronounced risk factor is a primary or acquired immunodeficiencies. Most prominently, the incidence of B-NHL is 70 times higher in patients infected by human immunodeficiency virus than in the general population [17]. Therefore, B-NHL is recognized nowadays as an AIDS-defining disease among human immunodeficiency virus infected patients [18]. Single nucleotide polymorphisms in IL-10 and TNF have been demonstrated to be associated with NHL, in particular diffuse large B-cell lymphoma [19], [20].

LILRB1 and LILRB2, which were two of the first members of the LILR family to be discovered, are the functionally best characterized LILRs. LILRB1 has a broad specificity for cytomegalovirus UL18 protein and different classical and non-classical HLA class I alleles [21], [22]. LILRB2 engagement has been demonstrated to inhibit activation of T-cells [23]–[25], B-cells and monocytes [5], [26], [27]. LILRA4 (ILT7), which is specifically expressed on plasmacytoid dendritic cells (pDCs) and associates with FcεRIγ, has been shown to bind to bone marrow stromal cell antigen 2 (BST2). BST2 mediated cross-linking of LILRA4 could inhibit TLR7/9 mediated activation of pDCs [7], [28]. LILRs thus have the potential to interact with different effector cells and participate in diverse aspects of the immune system.

The goal of this study was to determine whether *LILRA3* deletion might be associated with increased risk of malignant disease B-NHL, as well as identify the cells of the immune system that interact with LILRA3 and characterize the immune effects of LILRA3 in the immune responses.

## Materials and Methods

### Subjects

German caucasian patients diagnosed with various subtypes of B-NHL were included in the study (n = 196) obtained from the Clinic for Immunology und Rheumatology and the Department of Hematology, Hemostaseology, Oncology and Stem Cell Transplantation, Hannover Medical School. Patient characteristics and the proportion of the various subtypes of B-NHL are shown in Table 1. In addition, age- and gender-match healthy controls (n = 792) were obtained from the Institute for Transfusion Medicine, Hannover Medical School. The study was approved by the ethics committee of Hannover Medical School, with written informed consent from all patients.

**Table 1:** Patient characteristics.

mean age	64.5 (SD = 11.5) years
male:female	97∶100
B-NHL Subtype	Number
B-cell chronic lymphocytic lymphoma	24
Burkitt lymphoma	5
Diffuse large B-cell lymphoma	38
Follicular lymphoma	25
Hairy cell leukemia	9
Waldenström’s macroglobulinemia	11
Mediastinal large B-cell lymphoma	5
Mantle cell lymphoma	9
MALT lymphoma	6
Multiple myeloma	63
Primary CNS lymphoma	1

### Identification of LILRA3 Presence/Absence Variation

Genomic DNA was isolated from the blood, collected in EDTA collection tubes, using the QIAmp® DNA Blood Midi Kit (Qiagen, Hilden, Germany) according to manufacturer’s instructions. The polymerase chain reaction (PCR) with primers specific for the identification of the absence or presence of *LILRA3* and the subsequent agarose gel electrophoresis of the PCR product was performed as described previously [15]. Two-tailed Fisher’s exact test was used to compare the frequencies of the deletion (−/−) versus the non-deletion (+/+ and +/−) genotypes between the healthy controls and B-NHL patients, as well as to calculate the odds ratio.

### Antibodies

CD19-V450, CD8-V500, NKp46-APC, CD3-V500, CD80-FITC, CD86-PE, HLA-ABC-FITC were obtained from BD Biosciences (San Jose, USA). CD3-APC-Cy7 and GM-CSF-PE were from Biolegend (San Diego, USA). HLA-DR-PE was obtained from Beckman Coulter (Fullerton, USA).CD4-PE-DY747 CD14-FITC and CD14-PE were from Immunotools (Friesoythe, Germany).

### Isolation and Culture of Peripheral Blood Mononuclear Cells (PBMCs)

Heparinized human blood from healthy donors was obtained from the Institute of Transfusion Medicine, Hannover Medical School (Hannover, Germany). PBMCs were isolated via density gradient centrifugation using Biocoll (Biochrom, Berlin, Germany). For functional studies, PBMCs were cultured in growth medium at 37°C, 5% CO_2_. The PBMC culture medium comprised of RPMI 1640 medium supplemented with 2 mM L-Glutamin, 100 u/mL Penicillin, 100 µg/mL Streptomycin (all from Biochrom, Berlin, Germany) and 10% AB serum.

### Production of Recombinant LILRA3 and GAD-65 Using the Baculovirus System

The cDNA of LILRA3 and GAD-65 including a signal sequence and a C-terminal six His-tag was cloned into pVL1393. Sf9 cells (BD Biosciences) in Grace’s insect medium and 10% FSC for insect culture (both from Invitrogen, Carlsbad, USA) were transfected with pVL1393-LILRA3/GAD-65 using the BaculoGold Transfection Kit (BD Biosciences) in accordance with the manufacturer’s instructions. Virus was amplified at least three times in Sf9 cells and multiplicity of infection (MOI) was determined via plaque assay. For protein expression H5 cells (Invitrogen) were cultured in Express Five SFM medium (Invitrogen) at a concentration of 2×10^6^ cells/ml and infected with an MOI of 5. At day 4 post infection the supernatant was harvested and dialyzed against column buffer (50 mM NaH2PO4, 500 mM NaCl and 30 mM Imidazol) using an Äkta CrossFlow. Dialyzed supernatant was loaded onto a nickel affinity column (5 ml HisTrap, GE Healthcare, UK). Recombinant protein was eluted with 50% elution buffer (50 mM NaH2PO4, 500 mM NaCl and 400 mM Imidazol) and subsequently dialyzed against PBS, pH 7.4.

### Conjugation of Fluorochrome to Proteins

Recombinant LILRA3, GAD-65 and bovine serum albumin (BSA) (New England Biolabs, Frankfurt, Germany) were both conjugated via *N*-hydroxysuccinimide (NHS) chemistry using DyLight 488 (DL488) Antibody Labelling Kit (Pierce). Briefly, proteins were incubated with the NHS-activated DL488 fluorochrome (spectrally similar to Fluorescein-Isothiocyanate) for 60 minutes at room temperature in the dark. Excess unbound fluorochrome was subsequently removed by passing the protein-fluorochrome mixture through the supplied purification resin.

### Identification of LILRA3 Binding Cells Using Fluorochrome Conjugated LILRA3

PBMCs were incubated on ice for 30 minutes in PBS (Biochrom, Berlin, Germany) either without any blocking, or with blocking using 300 µg/mL of unlabelled LILRA3 or BSA. 6 µg of the respective fluorochrome-conjugated proteins were then added and allowed to incubate for another 30 minutes. CD19-V450, NKp46-APC, CD3, and CD14 were then added to stain for cell lineage. After 2 washes with PBS, the cells were fixed in 4% paraformaldehyde (PFA) and fluorescence determined using BD FACS Canto II. Results are expressed in terms of the mean fluorescent intensity of the FITC channel, as means of triplicates and analysed statistically using 2-way ANOVA with Bonferroni post test to compare every column.

### Mixed Lymphocyte Reaction Using ^3^H-thymidine Assay

In a 96 well plate, 10^5^ responder PBMCs were incubated with or without 10^4^ target allogeneic PBMCs, together with 0 to 500 ng/mL of LILRA3. After 6 days of incubation, the cells were pulsed with 0.4 µCi/well methyl-^3^H-thymidine (GE Healthcare, Chalfont St Giles, UK) and harvested 24 hours later with a harvester (Inotech, Dottikon, Switzerland). Radioactive uptake was measure using a microplate β-counter (PerkinElmer, Waltham, USA). Results are expressed in terms of counts per minute (cpm), as means of 3 wells, and statistically analysed using 1-way repeated measures ANOVA with Dunnett post test to compare to 0 ng/mL LILRA3 control.

### Mixed Lymphocyte Reaction Using Carboxyfluorescein Succinimidyl Ester (CFSE)

Responder PBMCs were stained for 15 minutes at 37°C with 0.25 µM CFSE in PBS, at a cell density of 10^6^ cells per mL. Excess CFSE was removed by incubating the stained cells in growth medium for 30 minutes at 37°C. In a 96 well plate, 10^5^ responder PBMCs were incubated with or without 10^4^ target allogeneic PBMCs, in the presence of 0 to 500 ng/mL LILRA3 or 500 ng/mL GAD-65. After 7 days of incubation, the cells were stained and fluorescence determined using BD FACS Canto II. Results are expressed in terms of percentage of cells that proliferated beyond the peak of non-proliferating cells, as means from 7 donors and analysed statistically using 2-way repeated measures ANOVA with Bonferroni post test to compare to 0 ng/mL LILRA3 control.

### Transwell Assay

CFSE-stained PBMC from each donor were divided into 2 groups. One group was untouched and the other was depleted of its CD14+ monocytes using anti-human CD14 Magnetic Particles (BD) according to the manufacturer’s protocol, selecting for the supernatant. In a 24-well suspension culture plate, 3×10^5^ CD14-depleted PBMCs (PBMC-MO) were pipetted into the wells (lower chamber) whereas an equal number of untouched PBMCs were pipetted into Thincert tissue culture inserts (upper chamber) (0.4 µM translucent, Greiner Frickenhausen, Germany). Irradiated allogeneic PBMCs were added either to the upper or lower chamber, in the absence or presence of 500 ng/mL LILRA3, and both chambers analysed for proliferation of the various cell subsets via flow cytometry after 7 days in culture. Results from 5 donors were statistically analysed using 2-way ANOVA with Bonferroni post test.

### Multiplex Cytometric Bead Array

Cell culture supernatants from previous CFSE MLRs were harvested and frozen. CBA Flex Sets (BD Biosciences) detecting IFN-α, IL-1β, IL-2, IL-4, IL-6, IL8, IL-10 and IL-12 were used to measure the cytokine concentrations in a multiplex assay. The effect of 500 ng/mL GAD-65 and 5 µg/mL GAD-65 purification flowthrough versus 500 ng/mL LILRA3 was also compared on the basis of IL-6 production in 6-day culture supernatants. The assay was performed according to the manufacturer’s instructions, fluorescence determined using BD FACS Canto II and analysed using the FCAP array software v1.0.1 (BD Biosciences). The detection limit, at the lowest concentration of our standard curve, was 9.6 pg/mL for each cytokine. Changes in concentrations were statistically analysed for each cytokine using 1-way repeated measures ANOVA with Tukey post test.

### Statistical Analysis

All statistical analyses were performed using GraphPad Prism 5 for Windows (GraphPad software Inc, San Diego, USA).

## Results

### Association of LILRA3 Deficiency with B-NHL

In order to determine whether *LILRA3* deficiency is associated with B-NHL, we performed dPCR to identify the *LILRA3* presence/absence genotype in each subject. Among the 196 B-NHL patients, 126 (64.3%) were homozygous for the presence variation, 57 (29.1%) were heterozygous and 13 (6.6%) were homozygous for the absence variation. Among the controls, the frequencies were 544 (68.7%), 224 (28.3%) and 24 (3.0%) respectively, which was in agreement with the Hardy Weinberg expectations (*X* ^2^ = 0.026; p = 0.87). The deletion genotype (−/−) was found to be significantly associated with B-NHL (p = 0.033; OR 2.27, 95% C.I. 1.136–4.551) (Table 2).

**Table 2:** Statiscal analysis of *LILRA3* deletion in B-NHL patients to controls.

	Healthy controls	B-NHL patients
**Number of cases**	**792**	**196**
*LILRA3* +/+	544 (68.7%)	126 (64.3%)
*LILRA3* +/−	224 (28.3%)	57 (29%)
*LILRA3* −/−	24 (3.0%)	13 (6.6%)
**Fisher’s exact test**		
p-value	0.033	
Odds ratio (95% C.I.)	2.27 (1.136–4.551)	

### The Binding Partner of LILRA3 is Expressed on Monocytes and B-cells

**Figure 1:**
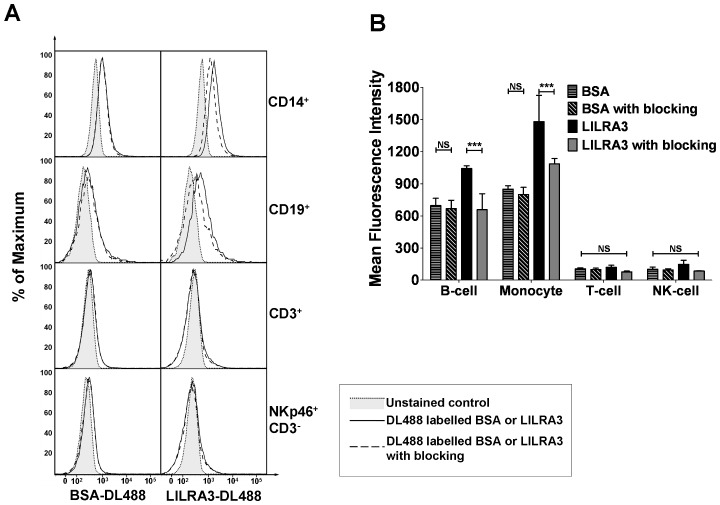
Binding of fluorochrome-conjugated LILRA3 to monocytes and B-cells. LILRA3 and BSA were conjugated with DL488 and used to stain PBMCs. (A) Representative histograms showing binding of LILRA3 or BSA, in the absence (no blocking) or presence (blocking) of excess unlabelled protein, to various PBMC cell subsets. (B) Triplicate analysis of the protein binding, expressed as the mean±SD of the MFIs, analysed using 2-way ANOVA with Bonferroni post test to compare every column. The experiment was duplicated in 2 further independent experiments which gave similar results. (***p<0.001, N.S.p>0.05).

We produced highly purified recombinant LILRA3 using a Baculovirus system. Recombinant LILRA3 was conjugated with DL488 and used to stain PBMCs by flow cytometry. It was found to bind significantly to both monocytes and B-cells, but not to T-cells or NK-cells (Figure 1). There was a higher level of binding on monocytes than on B-cells (MFI 1480 versus 1042), indicating a higher expression of the LILRA3 binding partner. When PBMCs were pre-incubated with an excess of unconjugated LILRA3 for competitive assays, the binding of the labelled LILRA3 was reduced to background levels (monocytes: MFI 1480 to 1085, p<0.001; B-cells: 1042 to 657, p<0.001), indicating that the binding and detection were specific. This experiment was reproducible in 2 further separate experiments (data not shown), with identical results. Control experiments with labelled GAD-65 did not show any specific binding to PBMCs which could be reduced in the presence of unlabelled GAD-65 (data not shown). 1 mg/mL of human intraveneous immunoglobulin and 100 µg/mL pan-HLA class I antibody W6/32 did not neutralize the staining of LILRA3 (Figure S2).

### LILRA3 has an Activating Effect on the Immune Response

**Figure 2:**
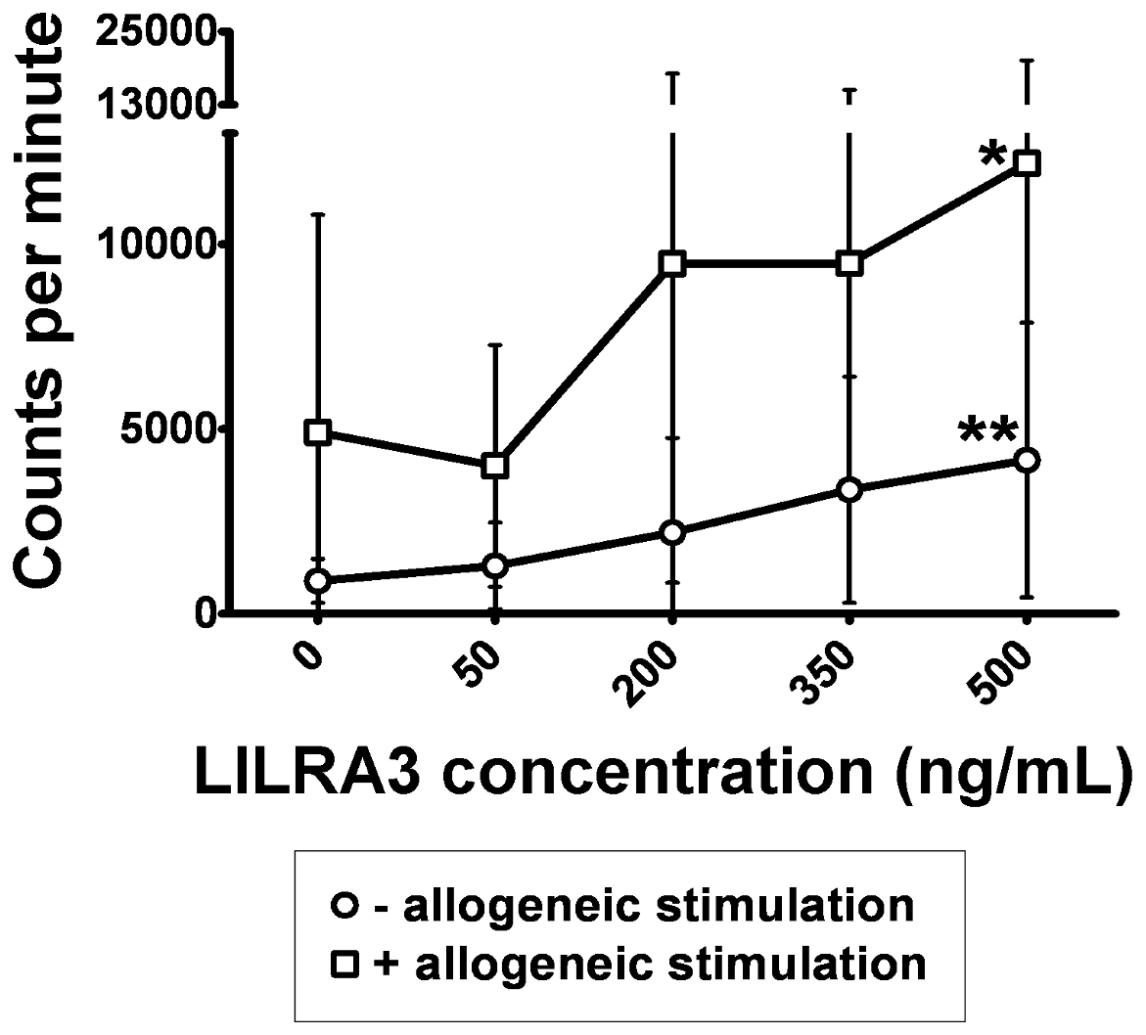
Induction of proliferation by LILRA3 in an MLR. Responder PBMCs were incubated in the absence or presence of suboptimal amounts of irradiated allogeneic feeder cells and varying concentrations of LILRA3, and the proliferation measured on the 7^th^ day with ^3^H-thymidine assay. Results expressed as mean±SD from 9 donors, statistically analysed using 1-way repeated measures ANOVA with Dunnett post test to compare to 0 ng/mL LILRA3 control. (*p<0.05, **p<0.01).

In order to deduce the general effect of LILRA3 in an immune reaction, we performed mixed lymphocyte reactions in the presence of varying concentrations of LILRA3, and measured the proliferation after 7 days with a ^3^H-thymidine assay. In the absence of additional allogeneic stimulation, there was a gradual increase in PBMC proliferation with increasing LILRA3 concentrations (Figure 2). More strikingly, in the presence of allogeneic stimulation, LILRA3 concentrations 200 ng/mL and above drastically increased the proliferative response. At 500 ng/mL, the increase in the proliferative response was much higher in the presence of allogeneic stimuli (12191 cpm, SD = 8120 cpm, mean difference to null LILRA3 = 7280 cpm, p<0.05) than in the absence thereof (4160 cpm, SD = 3720 cpm, mean difference to null LILRA3 = 3270 cpm, p<0.05).

### LILRA3 Stimulates the Proliferation of Cytotoxic T-cells and NK Cells

**Figure 3:**
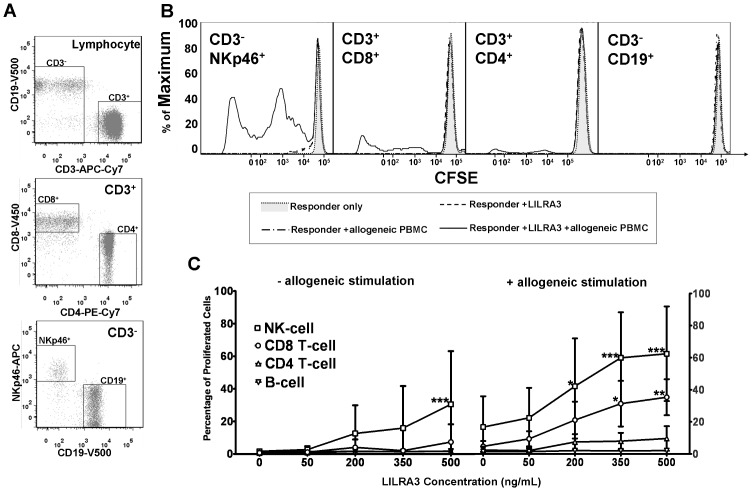
LILRA3 induces proliferation of NK-cells and cytotoxic CD8^+^ T-cells. CFSE stained responder PBMCs were used in an MLR to identify the cell population that was expanding. On the 7^th^ day of incubation with varying LILRA3 concentrations, PBMCs were stained to identify the various cell subsets by flow cytometry. (A) Among the leukocytes, CD3^+^ and CD3^−^ populations were segregated. In the CD3^+^ population, the CD4^+^CD8^−^ and CD8^+^CD4^−^ T-cell populations were gated whereas in the CD3^−^ population, the CD19^+^NKp46^−^ B-cell and NKp46+CD19- NK-cell populations were gated and analysed for CFSE proliferation. (B) Representative histograms depicting the proliferation of the NK-cells (CD3^−^NKp46^+^), CD8 T-cells (CD3^+^CD8^+^), CD4 T-cells (CD3^+^CD4^+^) and B-cells (CD3^−^CD19^+^) by their CFSE fluorescence. The percentage of cells which had proliferated was obtained by gating to the left of the peak of unproliferated cells in unstimulated responders. (C) Percentage of proliferated cells in various cell subsets induced by LILRA3 and MLR response. Results expressed as mean±SD from 5 donors, statistically analysed using 2-way repeated measures ANOVA with Bonferroni post test to compare to 0 ng/mL LILRA3 control. (*p<0.05, **p<0.01, ***p<0.001).

Since LILRA3, through binding to cells expressing CD19 or CD14, was able to induce proliferation in a MLR, we next determined the expanding cell population. We incubated CFSE labelled PBMCs together with LILRA3 and/or suboptimal levels of allogeneic feeder cells, and determined the percentage of various lymphocyte subsets that proliferated after 7 days via flow cytometry. There were considerable variations in the strength of the proliferation response between individuals, but the general trend could be observed when we pooled the data of 5 donors. Similar to the 3H-thymidine assay, LILRA3 was able to induce a weak proliferation of lymphocytes, which became much more prominent in the presence of allogeneic stimuli. This proliferation was specific to LILRA3 because similar experiments performed with glutamate decarboxylase-65 (GAD-65), which is an autoantigen in type-1 diabetes but should be an immunologically irrelevant protein in healthy donors, did not induce a proliferation in CFSE-stained leukocytes (Figure S1A). In the absence of allogeneic stimulation, proliferation of NK-cells could be observed to increase gradually with increasing LILRA3 concentrations, which at 500 ng/mL LILRA3 was 30.5% (SD = 32.6%, mean difference to null LILRA3 = 28.9%, p<0.001) (Figure 3C, left panel). In the presence of allogeneic stimulation, there was a more striking increase in the proliferation of both NK-cell and CD8 T-cell, which at 500 ng/mL LILRA3 was 62.4% (SD = 29.5%, mean difference to null LILRA3 = 45.7%, p<0.001) and 35.3% (SD = 4.7%, mean difference to null LILRA3 = 30.6%, p<0.001) respectively (Figure 3C, right panel). The effect of LILRA3 on the proliferation of CD4^+^ T-cells was at best modest, which in the presence of allogeneic stimulation and 500 ng/mL LILRA3 was 9.6% (SD = 7.7%, mean difference to null LILRA3 = 7.2%, p>0.05). There was no observable effect on the proliferation of CD19^+^ B-cells.

### LILRA3 Stimulated Proliferation of Lymphocytes Depends on Both Cell Contact to and Soluble Factors Secreted by Monocytes

**Figure 4:**
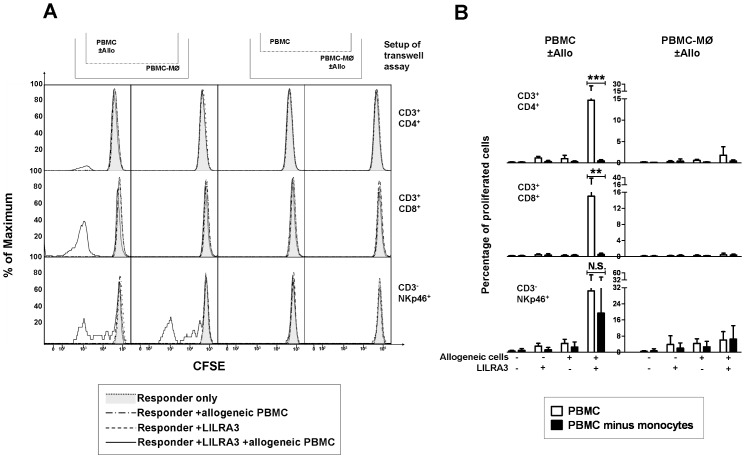
LILRA3 mediated proliferation is dependent on monocytes. T-cell proliferation is dependent on cell-cell contact to monocytes whereas NK-cell proliferation is dependent on soluble factors. In a transwell chamber, using CFSE-stained PBMCs, whole PBMCs were filled in the upper chamber and PBMCs-MO in the lower chamber. In the absence or presence of 500 ng/mL LILRA3, irradiated allogeneic feeder PBMCs were added to either chambers. The proliferation of the various cell subsets was determined by flow cytometry after 7 days. (A) Representative histogram plots showing proliferation of CD4 T-cells (CD3^+^CD4^+^), CD8 Tcells (CD3^+^CD8^+^) and NK-cells (CD3^−^NKp46^+^) indicated by their CFSE fluorescence. The percentage of cells which had proliferated was obtained by gating to the left of the peak of unproliferated cells in unstimulated responders. The pictogram above the histograms shows whether the column of histograms belong to the upper chamber or lower chamber, and whether the allogeneic stimulation (+allo) was given to whole PBMCs or PBMCs-MO (B) Statistical analysis of results from 4 donors expressed as mean±SD, performed using 2-way ANOVA with Bonferroni post test. (**p<0.01, ***p<0.001, N.S.p>0.05).

Given that LILRA3 is likely to bind to specific receptors on monocytes, but then induces proliferation of the cytotoxic lymphocytes, we hypothesize that this LILRA3-induced proliferation was due to the monocytic activation of either their antigen presenting capabilities and/or cytokine production. In order to test whether monocytes might be the primary cell population through which LILRA3 acts, and whether the proliferation could be induced primarily through cell-cell contact or cytokines, we devised a transwell assay, consisting of 2 compartments of either CFSE-labelled whole PBMC or CFSE-labelled PBMC depleted of CD14^+^ monocytes (PBMC-MO). In the presence or absence of LILRA3, either of both compartments was incubated with allogeneic PBMCs. The percentage of cells that proliferated in both compartments after 7 days was determined. In accordance with the previous proliferation assays, in the transwell system, the propensity for proliferation in whole PBMCs was only substantial when both LILRA3 and allogeneic stimuli were present (Figure 4B, graphs on the left). However, the proliferation was abrogated in monocyte depleted PBMCs (Figure 4B, graphs on the right), demonstrating the importance of the role of monocytes in LILRA3 mediated activation and proliferation of lymphocytes. Even if the lymphocytes were in direct contact to the allogeneic PBMCs, in the absence of monocytes, LILRA3 could not induce a proliferation. Looking at both CD4^+^ and CD8^+^ T-cells, their proliferation in doubly stimulated whole PBMCs in the upper chamber did not translate into a propensity for proliferation in the monocyte deprived lower chamber, even if they were in the same well and shared the same cytokine milieu (Figure 4B, graphs on the left). The proliferation between the whole PBMCs and PBMC-MO chambers was decreased in CD4^+^ T-cells from 14.6% (SD = 11.0%) to 0.5% (SD = 0.3%, p<0.001), and in CD8^+^ T-cells from 15.0% (SD = 23%) to 0.6% (SD = 0.3%, p<0.01), implying that the proliferation of the T-cells depended on direct cell contact to monocytes. On the other hand, the proliferation of NK cells in the upper chamber (30.7%, SD = 24.5%) was correlated with proliferation in the lower chamber (19.6%, SD = 27.1%, p>0.05), indicating that the LILRA3 induced proliferation of NK-cells depended on cytokines or soluble factors and not cell-to-cell contact.

### Upregulation of Various Proinflammatory Cytokines by LILRA3

**Figure 5:**
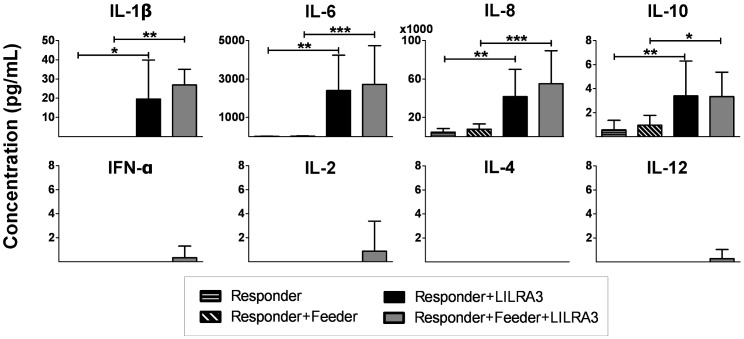
Cell culture supernatants from previous CFSE proliferation experiments were harvested and used to determine concentrations of various cytokines via a multiplex bead array. Results expressed as mean±SD from 8 donors, statistically analysed for each cytokine using 1-way repeated measures ANOVA with Tukey post test. (*p<0.05, **p<0.01, ***p<0.001).

Since soluble factors were responsible for a part of the proliferation response induced by LILRA3, we screened for differentially regulated cytokines using a multiplex bead array assay for cytokines, which we suspected to impact the activation and/or proliferation of the effector cytotoxic lymphoytes. Cell supernatants harvested from the proliferation assays were analysed and the concentrations of 8 different cytokines were measured, resulting in the detection of 4 significantly upregulated cytokines (Figure 5). In the presence of 500 ng/mL LILRA3, the two cytokines that were conspicuously upregulated were IL-8 (from 4.65±3.78 to 41.5±28.4 ng/mL) and IL-6 (from 14.5±8.03 to 2400±1830 pg/mL), followed by IL-1β (from 0 to 19.6±20.4 pg/mL). IL-10 was only weakly, but significantly, upregulated (from 0.57±0.79 to 3.39±2.91 pg/mL). This was below the concentration of our lowest standard (9.6 pg/mL) but above the theoretical detection limit (0.13 pg/mL) Control experiments with GAD-65 did not induce any upregulation of IL-6 (Figure S1B). To our surprise, even though we observed significant proliferation of T-and NK-cells at 500 ng/mL of LILRA3 (Figures 2 and 3), cytokines having an impact on T-and NK-cell differentiation and proliferation, such as IL-2, IL-4 and IL-12, as well as IFN-α, were not significantly upregulated in the supernatants.

## Discussion

We show now for the first time that *LILRA3* deletion is associated with the malignant disease B-NHL, whose etiology still remains mostly elusive, with only 2 genetic variations in the form of single nucleotide polymorphisms in TNF and IL-10 credited to be genetic risk factors [19], [20]. Interestingly, a history of various autoimmune diseases, including rheumatoid arthritis (RA), systemic sclerosis and Sjögren’s syndrome, has been linked to an increased risk of NHL [29] and previous studies from our group has also observed a link between *LILRA3* deletion and MS and Sjögren’s syndrome [15], [16]. Within our cohort of B-NHL patients, it may be interesting to note that of the patients deleted for *LILRA3*, 1 out of 13 (7.7%) patients also suffers from Sjögren’s syndrome and RA each, whereas among the patients sufficient for *LILRA3*, only 1/175 (0.0057%) suffers from Sjögren’s syndrome and 5/175 (2.9%) from RA (Table 3).

**Table 3:** Prevalence of autoimmune conditions among the B-NHL patients.

Genotype/AutoimmuneDisorder	+/+(n = 120)	+/−(n = 55)	−/−(n = 13)
Arthritis	5	–	1
Sjögrens Syndrome	–	1	1
Psoriasis	4*	–	–
Autoimmune hemolytic anemia	3*	–	–
Autoimmune Thyroiditis	4	2	–
Sarkoidosis	–	1	–
Vaskulitis	1	–	–
Chronic Inflammatory Bowel Disease	1	–	–
Axial Spondyloarthritis	1	–	–
Total	18*	4	2
%	15.0	7.3	28.6

*A patient had both psoriasis and autoimmune hemolytic anemia.

Although our study was able to demonstrate an association for the deletion of *LILRA3* with B-NHL as a whole, our cohort was too small to be sufficiently powered to analyze the association with the different subtypes of B-NHL and a larger sized cohort for the various subtypes should be studied. There is a huge potential for elucidating the role of *LILRA3* deletion in the etiology in B-NHL, either as a direct causative agent for B-NHL or as a cause of an increased susceptibility to virus infections which in turn may increase the risk for B-NHL.

Although LILRA3 was able to induce by itself some proliferation in the NK-cells, the proliferation was most robust in the presence of an additional allogeneic stimulation, leading to even greater proliferation of NK-cells, as well as proliferation of CD8 T-cells, both cytotoxic cell subsets. This hints towards the role of LILRA3 as a conditional immunomodulator.

LILRA3 mRNA has been shown to be strongly expressed towards the end stages of dendritic cell differentiation, as well as in osteoclasts [30]. Since we have demonstrated that LILRA3 was able to bind to a specific receptor on monocytes and B-cells, which are both antigen presenting cells, it is tempting to suggest that LILRA3 acts on an antigen presentation level.

We also saw a conspicuous upregulation of proinflammatory cytokines IL-8 and IL-6, and to a lesser extent IL-1β, when PBMCs were incubated with LILRA3. Although IL-10 was also slightly upregulated, its concentration pales considerably when compared to the other upregulated cytokines. Thus rather than playing an anti-inflammatory role, IL-10 probably has a more modulating role in LILRA3 mediated response. Taken together in light of our findings, we suggest that LILRA3 serves to either prime leukocytes for or amplify an ongoing cytotoxic response.

Recently, Ryu et. al. showed using surface plasmon resonance that LILRA3 could bind to both classical and non-classical HLA class I molecules albeit at a lower affinity compared to LILRB1 and LILRB2 [31]. However, we could not detect a neutralization of our LILRA3 binding with pan HLA-class I neutralization antibody W6/32 (Figure S2). This could be either because the binding we observed was not attributed to HLA class I molecules, or thatthe W6/32 antibody did not bind to epitopes recognized by LILRA3. Jones et. al. demonstrated that LILRA3, as well as LILRA1, displayed preferential binding for the HLA-C molecule, especially the free heavy chain and that LILRA3 and LILRA1 had strongly correlating HLA class I binding profiles, possibly sharing highly similar HLA class I recognition sites [32]. MHC I. CD14^+^ monocytes have been demonstrated to express FHCs at significantly higher levels in comparism to the other PBMC subsets, although no expression of free heavy chains on CD19^+^ B-cells could be seen [33]. Therefore, the question of whether the binding of LILRA3 to free heavy chains could explain the immunomodulatory consequences we have seen begs to be answered.

Considering that the deletion of LILRA3 is associated with both ends of the spectrum of immune dysregulation, and that MS, pSS and B-NHL have all been discussed to be triggered by virus infection, it remains to be seen what effect LILRA3 has in the course of a virus infection. Since LILRA3 has such a proliferative effect on the cytotoxic cells, it could accelerate the clearance of viruses and other intracellular pathogens whose clearance require mainly the cytotoxic response. Also, in the case of B-NHL, *LILRA3* deletion could have direct impact on the pathogenesis of B-NHL, since its deletion would imply an impaired cytotoxic effector response, which is important for tumor clearance. On the other hand, in the case of an established chronic inflammation, the cytokine burst produced by LILRA3, in particular of IL-1 and IL-6, could exacerbate the course of the disease and lead to increased pathology. Additionally, it has long been acknowledged that there is a link between autoimmune and lymphoproliferative diseases, thus it remains to be seen if LILRA3 plays a role in the underlying mechanisms connecting both ends of the spectrum.

Since we have shown that LILRA3 possesses a myriad of potent immunomodulatory properties, and that its deletion is a risk factor for some diseases of the immune system, exploration of the use, or neutralization, of LILRA3 as a therapeutic agent would be of major clinical relevance.

In conclusion, soluble LILRA3 directly activates monocytes and B cells, which in turn stimulate T- and NK-cell proliferation. The deletion of *LILRA3* is therefore expected to cause an impairment of the immune defense against tumors, in line with the significant association of *LILRA3* deletion with B-NHL observed in our study.

## Supporting Information

Figure S1 **Control protein GAD-65 and supernatant flowthrough from protein purification does not induce any activation as compared to LILRA3.** (A) CFSE stained responder PBMCs were used in an MLR with 500 ng/mL each of LILRA3, GAD-65 and flowthrough from the purification of GAD-65. Result from triplicates shown as mean±SEM obtained from 1 donor, statistically analysed using 1-way ANOVA with Dunett post test. The experiment was reproduced in 1 further donor. (B) IL-6 concentration was measured in supernatants obtained from 6 days incubation with 0.5 µg/mL LILRA3, 0.5 µg/mL GAD-65 and 5 µg/mL supernatant flowthrough from the purification of GAD-65. Although LILRA3 induced a strong upregulation of IL-6, GAD-65 and its flowthrough did not stimulate any significant production of IL-6. Results shown as mean±SD from 4 donors, statistically analyzed using 1-way ANOVA with Dunett post test. (**p<0.01, ***p<0.001).
(TIF)

Figure S2 **DL488-Labelled LILRA3 was used to stain for CD14^+^ monocytes and CD19^+^ B-cells in the presence of 77 and 135 µg/mL unlabelled LILRA3, as well as >1 mg/mL human IVIG and 100 µg/mL pan-HLA class I neutralization antibody W6/32.** Results shown as mean±SEM from 1 donor, statistically analyzed using 1-way ANOVA with Dunett post test. The experiment was reproduced in 1 further donor. (**p<0.01, ***p<0.001).
(TIF)
