# Supplementary figures and images for: Association of the LILRA3 Deletion with B-NHL and Functional Characterization of the Immunostimulatory Molecule
Source: PLoS One. 2013 Dec 9;8(12):e81360. doi: 10.1371/journal.pone.0081360 (PMC3867304; doi:10.1371/journal.pone.0081360)

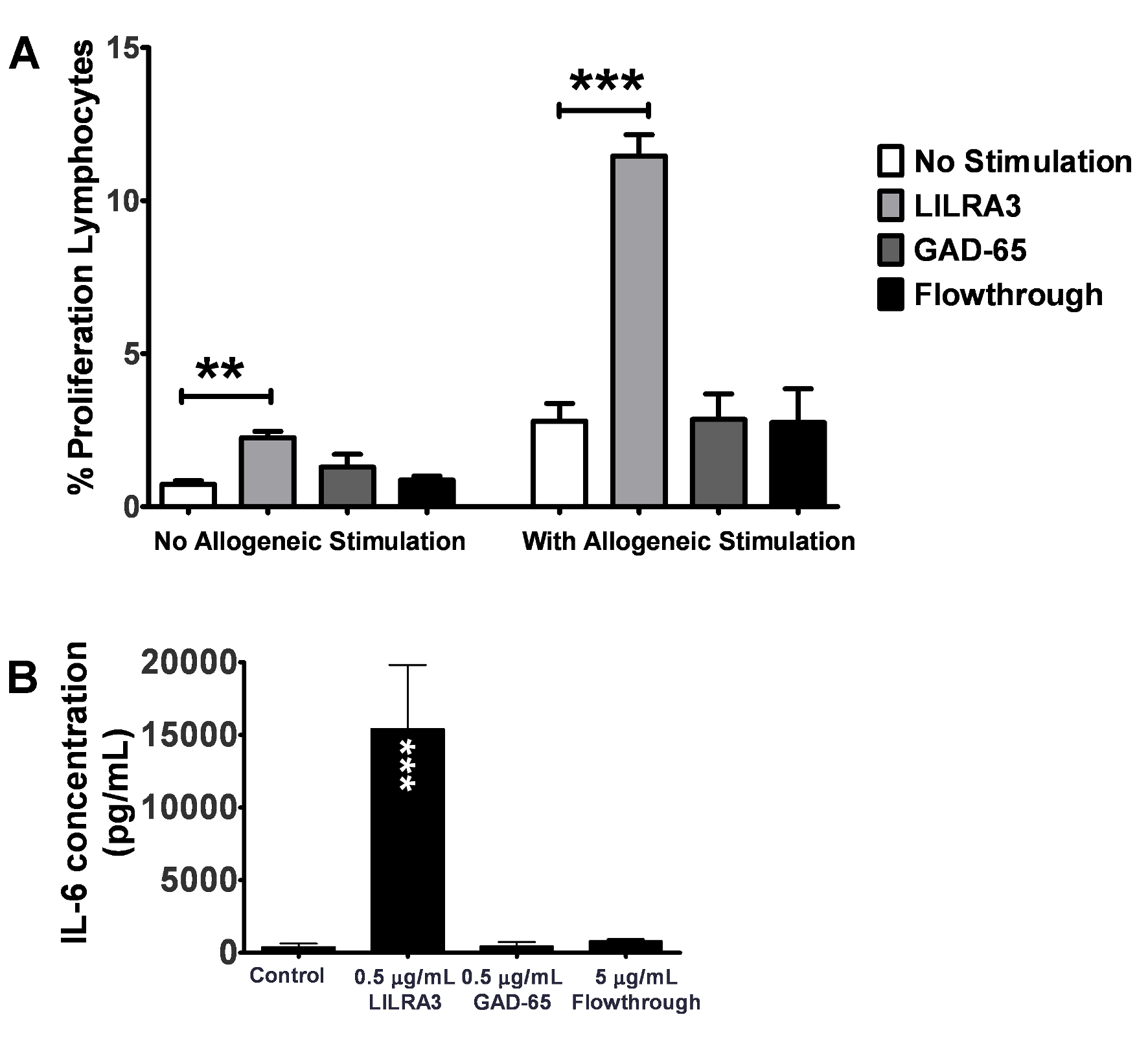

Supplement: Figure S1 — Control protein GAD-65 and supernatant flowthrough from protein purification does not induce any activation as compared to LILRA3. (A) CFSE stained responder PBMCs were used in an MLR with 500 ng/mL each of LILRA3, GAD-65 and flowthrough from the purification of GAD-65. Result from triplicates shown as mean±SEM obtained from 1 donor, statistically analysed using 1-way ANOVA with Dunett post test. The experiment was reproduced in 1 further donor. (B) IL-6 concentration was measured in supernatants obtained from 6 days incubation with 0.5 µg/mL LILRA3, 0.5 µg/mL GAD-65 and 5 µg/mL supernatant flowthrough from the purification of GAD-65. Although LILRA3 induced a strong upregulation of IL-6, GAD-65 and its flowthrough did not stimulate any significant production of IL-6. Results shown as mean±SD from 4 donors, statistically analyzed using 1-way ANOVA with Dunett post test. (**p<0.01, ***p<0.001). (TIF) [file pone.0081360.s001.tif]

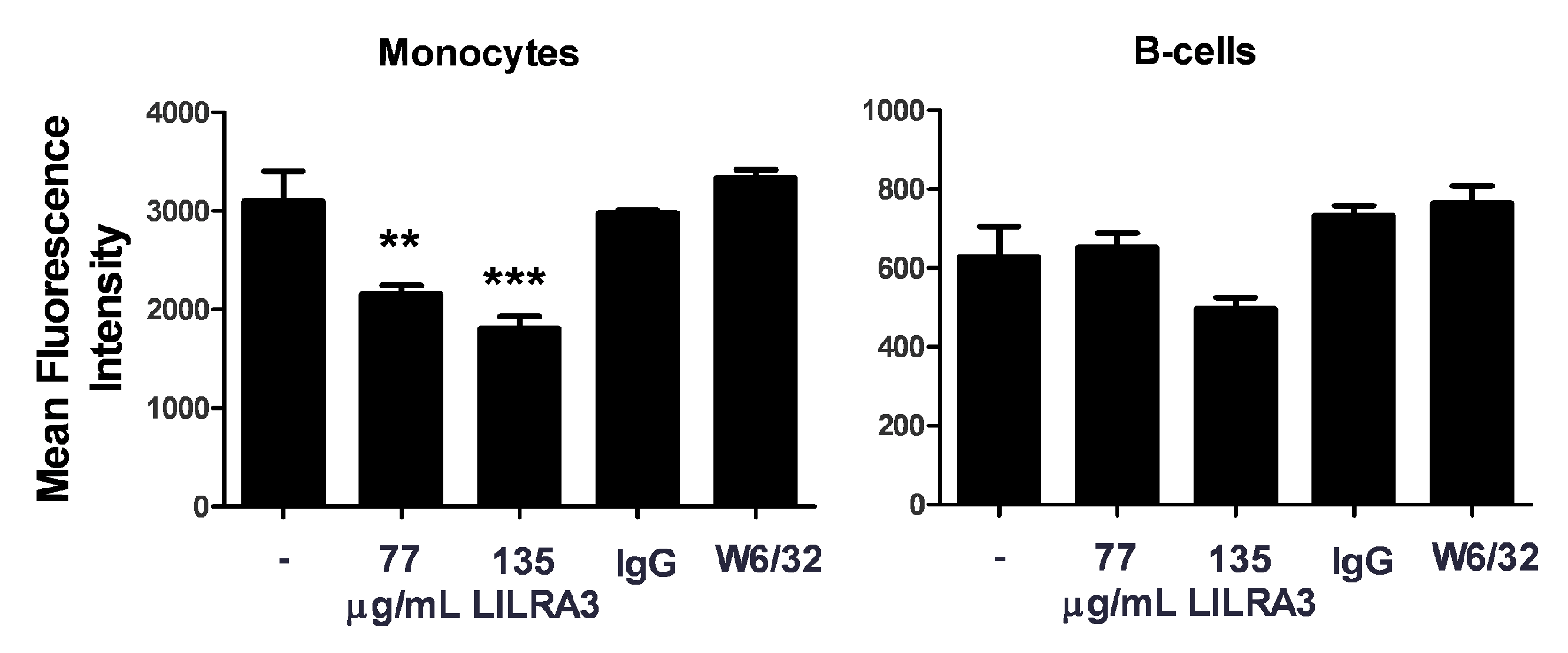

Supplement: Figure S2 — DL488-Labelled LILRA3 was used to stain for CD14+ monocytes and CD19+ B-cells in the presence of 77 and 135 µg/mL unlabelled LILRA3, as well as >1 mg/mL human IVIG and 100 µg/mL pan-HLA class I neutralization antibody W6/32. Results shown as mean±SEM from 1 donor, statistically analyzed using 1-way ANOVA with Dunett post test. The experiment was reproduced in 1 further donor. (**p<0.01, ***p<0.001). (TIF) [file pone.0081360.s002.tif]
